# The Role of Family Physicians in Children’s Sleep

**DOI:** 10.7759/cureus.65131

**Published:** 2024-07-22

**Authors:** Cátia S Brito, Oleksandra Umanets, Diana Silva, Duarte Santos, Jéssica Santos

**Affiliations:** 1 Family Medicine, Unidade de Saúde Familiar (USF) Arandis - Unidade Local de Saúde (ULS) Oeste, Torres Vedras, PRT; 2 Family Medicine, Unidade de Saúde Familiar (USF) Gama - Unidade Local de Saúde (ULS) Oeste, Torres Vedras, PRT; 3 Family Medicine, Unidade de Saúde Familiar (USF) São Sebastião - Unidade Local de Saúde (ULS) Oeste, Torres Vedras, PRT

**Keywords:** sleep recommendations, primary healthcare, sleep disorders, sleep assessment, childhood sleep

## Abstract

Sleep holds significant importance for maintaining health and aiding in illness recovery. Its deprivation impacts all human organ systems, from cognitive function, social interaction, and work capacity to cellular regeneration and immune function. Therefore, sleep plays a crucial role in our body and maintaining health and well-being.

Given its importance and close relationship with the neurodevelopment and growth of children and adolescents, this topic is highly significant in pediatric and adolescent health consultations. Family physicians, due to their proximity and understanding of the individual within their family, have the prerogative to contribute to family literacy, empower them, and significantly enhance the quality of life and overall health.

## Introduction and background

Sleep (derived from the Latin word somnus) is a physiological state characterized by sensory insensitivity and the need for rest [1]. It is characterized by periodicity, with a temporary suspension of perceptual-sensory and voluntary motor activity [2]. It presents various degrees of depth, in which there is a greater or lesser capacity to provoke awakening, reflected in the alteration of brain electrical activity, which can be more or less intense, and also by mental activity, namely dreaming [3].

There are five distinct stages of sleep that can be measured by polysomnography: the rapid eye movement (REM) stage and four non-rapid eye movement (NREM) sleep stages [3]. It is during REM sleep that most dreams occur, representing about 80% of sleep in newborns and about 20% in adults [3]. The most accepted theory regarding the function of sleep explains that it allows changes in brain structure and organization, referred to as "brain plasticity" [4].

The primary objective of this review is to compile scientific evidence on approaches to children's sleep, as this is a controversial topic and it is difficult to conduct complex studies on the subject. Therefore, we have gathered information on approaches to sleep, the main sleep disorders, and their management, emphasizing the role of the family doctor in evaluation and guidance.

## Review

Methods

The following scientific databases were searched: British Medical Journal, Evidence-Based Medicine Online, National Guideline Clearinghouse, National Library of Guidelines, The Cochrane Library, DARE, MEDLINE/Pubmed, and Index of Portuguese Medical Journals, for articles from 2000 to March 2024, using the keywords infant sleep, sleep assessment, sleep disorders, primary health care, sleep disorders, sleep strategies for children, and family doctor. Articles were selected based on the relevance of their abstracts to the aim of this study and were available in English, Portuguese, and Spanish.

Sleep in early childhood and its importance

Despite significant advances in the field of neuroscience, the functions of sleep are not yet fully understood [5]. The currently most accepted theory is that sleep is related to processes of brain plasticity involved in the maturation of the nervous system, memory consolidation, and learning [6-7].

Sleep habits are influenced by various factors, including biological, psychological, environmental, familial, sociocultural, and developmental stages [6]. On the other hand, sleep influences the regulation of homeostatic and hormonal systems underlying somatic growth, maturation, and bioenergetics [6].

Thus, it is evident that sleep represents a crucial daily activity for growth and development, with even greater importance for younger individuals who demonstrate a greater need for sleep time for their proper development [5,6,7]. Therefore, when evaluating a child, it becomes crucial that the approach be holistic, taking into account their family and social context, daily activity, including dietary habits and regular physical activity, along with the assessment of the quality and quantity of sleep [7].

Daily reference values for sleep duration

The duration of sleep is an important factor that will also influence its quality [5,6]. Daily sleep time encompasses nighttime sleep and naps that occur during the day [7,8]. Nap time decreases as the child grows into adulthood [8]. The reference values for total sleep time and naps are represented in Table 1, noting that they are average values with interpersonal variability [8].

**Table 1:** Sleep time in each age [8]

Age	Total recommended time
≤3 months	14-17 hours
4-11 months	12-15 hours
12-24 months	11-14 hours
3-5 years	10-13 hours
6-13 years	9-11 hours
14-17 years	8-10 hours
>18 years	7-9 hours

Studies on sleep

Most existing studies focus on inadequate sleep problems, allowing us to infer about its functions [7]. Aspects are subject to subjective evaluation through questionnaires as well as more objective methods (e.g., polysomnographic study and actigraphy) [7]. The majority of experimental studies on the effects of sleep deprivation have been conducted on animal models and adult humans, with conclusions that are difficult to extrapolate to childhood ages, as children and adolescents present various peculiarities: they are still developing, the structure and duration of sleep are not the same as in adults, and the manifestation of sleep deprivation is also different [5-7]. Given the difficulty of conducting studies at an early age, primarily due to ethical issues, most studies in this age group are observational, making it often difficult to establish causal relationships and eliminate possible bias [7]. Additionally, the interpretation of results obtained in sleep studies and their consequences are challenging to objectify due to the multiplicity of systems affected [6-7].

Consequences of sleep deprivation

Inadequate sleep, whether in quantity (duration) or quality (neurophysiological structure), or both, results in deleterious effects at various levels [6,9-18]: Cognitive functions, such as attention and memory, are altered, and there is a slowing in stimulus processing. Higher cognitive functions, including abstraction, creativity, and problem-solving ability, are also affected. Emotionally, inadequate sleep can lead to feelings of fatigue, lack of vigor, irritability, and behavioral apathy. There is an increased risk of accidents, a significant cause of morbidity and mortality in pediatric ages, with several studies finding a relationship between sleep deprivation and accidents, concluding that sleep deprivation increases the risk of accidents. Additionally, daytime sleepiness, symptoms mimicking attention deficit hyperactivity disorder, and metabolic changes, such as obesity, have been associated with sleep deprivation. Although the mechanisms are not fully understood, further studies are required to understand the link between sleep deprivation and obesity. Inadequate sleep also influences the normal growth and development of children and negatively impacts family dynamics, disrupting the sleep of other family members and affecting the physical and psychological well-being of the entire family.

Due to the impact that this essential activity for human life has on the well-being of children and their caregivers, it is of utmost importance to understand sleep habits in this specific population as well as to identify any potential problems [6,7,12,17,18].

Most common sleep problems

According to the Children and Sleep National Sleep Foundation, up to 30% of children aged two to five years and 15% of school-aged children have trouble falling asleep or sleeping through the night regularly. Less than one-third of teenagers get enough sleep, according to a survey by the Centers for Disease Control and Prevention [19]. Children rarely complain about these problems/disruptions, which are commonly reported by parents/caregivers [6]. As mentioned earlier, children's sleep problems are habits, behaviors, and/or sleep patterns that are undesirable and will affect the entire family dynamic [6,20]. Table 2 presents the most common disturbances, according to the classification of the International Classification of Sleep Disorders third edition (ISCD-3) [20].

**Table 2:** Most common disturbances according to the ISCD-3 classification NREM: non-rapid eye movement, REM: rapid eye movement, ISCD-3: International Classification of Sleep Disorders third edition [20]

Major categories	Most common causes/problems
Insomnias	Chronic insomnia
	Inadequate sleep hygiene
Parasomnias	NREM-related parasomnias (e.g., confusional arousals; sleep terrors; sleepwalking)
	REM-related parasomnias (e.g., nightmare disorder)
	Other parasomnias (e.g., sleep enuresis)
	Isolated symptoms (e.g., sleep talking)
Circadian rhythm sleep disorders	Delayed sleep phase type
	Irregular sleep-wake type
Sleep-related breathing disorders	Obstructive sleep apnea, pediatric
	Primary sleep apnea of infancy
	Apnea of prematurity
Sleep-related movement disorders	Restless legs syndrome
Hypersomnias of central origin	
Other sleep disorders	

According to the Clinical Practice Guide on Sleep Disorders in Childhood and Adolescence in Primary Care of the Spanish National Health Service, in order to facilitate management in primary healthcare, sleep disorders in these age groups can be grouped into three categories [20]: (1) difficulty falling asleep: insomnia due to inadequate sleep hygiene, behavioral insomnia, restless legs syndrome, and delayed sleep phase syndrome; (2) abnormal events during the night: obstructive sleep apnea/hypopnea syndrome, sleepwalking, night terrors, confusional arousals, nightmares, and sleep-related rhythmic movement disorders; (3) and daytime sleepiness: chronic sleep deprivation of multifactorial origin and narcolepsy.

Assessment of children’s sleep

Children's sleep can be studied in a subjective or objective manner [7,20]. Anamnesis is extremely important as it allows the gathering of relevant data that provides the healthcare professional with a comprehensive and integrative view of the child and their family [20]. It should be as complete as possible and encompass the current history, personal and family backgrounds, habits, routines, and socio-educational path [20].

A subjective study is based on questionnaires, usually retrospective, conducted by parents or the children themselves [20-22]. Table 3 presents some of the possible questionnaires for subjective evaluation [20-22].

**Table 3:** Questionnaires for sleep evaluation [20-22]

Questionnaires for Sleep Evaluation
Brief Infant Sleep Questionnaire (BISQ)
Children's Sleep Habits Questionnaire – Portuguese version (CSHQ-PT)
Sleep Self Report (Children)
Modified Pediatric Epworth Sleepiness Scale
BEARS Questionnaire (Bedtime, Excessive daytime sleepiness, Awakening during the night, Regularity and duration of sleep, Snoring)
Sleep Disturbance Scale for Children (SDSC)
Sleep Disorders Inventory for Students
Pediatric Sleep Questionnaire
Van Dream Anxiety Scale
Nightmare Effect Survey
Nightmare Distress Questionnaire

In a family physician’s clinical practice, the BEARS questionnaire can be used as a screening tool in children over two years of age in order to identify sleep problems early during child health surveillance consultations [22]. It is also possible to assess sleep through sleep diaries [20]. This is another tool that allows awareness of the number of hours of sleep and reflection on them for positive or negative consequences during the day (e.g., daytime sleepiness as a negative consequence) [20]. This type of assessment generally leads to greater adherence to intervention [20]. Objectively, there are two ways to study sleep: polysomnography and actigraphy [20-22].

Strategies that can help solve some issues

The type of strategy/intervention will depend on the underlying problem that is diagnosed, the child's age, and the reality of each family [20]. It is extremely important that the chosen strategy is in harmony with the family and that they are actively involved in the decision-making process, with each intervention being adjusted to each context to maximize its success [20].

According to age, it is necessary to ensure that sleep time and quality are respected [22]. As described above, sleep time depends on the age group, and it is important to ensure good development for the child [22]. During the first year of life, the strategy sometimes involves managing parents' expectations, reassuring them, and informing them [23,24]. At one to two months of age, circadian activity begins to develop, and by three to four months, babies already have stable melatonin production [23]. Between six and nine months, wakefulness increases, naps are well established, and by 12 months, 70% to 80% of babies mainly sleep at night [23]. Therefore, in the first six months of life, it is normal for night awakenings to occur, with a distribution that follows the sleep cycle (which lasts 90-120 minutes) and occurs more commonly in the REM stage [23]. In these cases, it is common for the child to return to sleep spontaneously [23]. It is important to raise awareness among parents about the normal functioning of babies and encourage the adoption of pre-sleep routines and rituals that work for the family to ensure good rest for everyone [20,22]. It is also important to analyze the baby's sleeping environment, which should be in a calm and clean environment with a good temperature [20,22]. The mattress on which the baby sleeps should be firm and not inclined, and the bedding should not exceed the nipple line, ensuring it does not overheat the baby [20,22]. For children, it is also necessary to ensure that the room is suitable, pleasant, without technology, and not associated with anything negative, such as "punishment” [20].

Table 4 presents a summary of possible treatments/strategies, excluding pharmacological treatments, according to the type of pathology [21,23]. Behavioral interventions for pediatric sleep problems (such as parent education and positive bedtime routines), especially in young children, have been shown to produce clinically significant improvements [21]. This is particularly important given the relative lack of data on the use of pharmacological interventions for sleep difficulties in pediatrics, which will not be addressed in this article.

**Table 4:** Resume table of treatments/possible strategies according to pathology type (exclusion of pharmacological treatment) [21,23]

Sleep disorder	Examples	Treatment
Insomnia	Inadequate sleep hygiene, stimulating activities, consumption of stimulants, environment, disorganized sleep practices, psychophysiological insomnia, behavioral insomnia of childhood Insomnia symptoms for more than three months with associations that inhibit sleep (common since school age)	Sleep hygiene, regular schedules, routines, physical activity, reduction of screen time, reduced consumption of stimulants, adequate environment to sleep, cognitive behavioral therapy
			NREM parasomnias	Confusional arousals, sleep terrors, sleepwalking	Do not interfere, it is always important to a good sleep hygiene. If necessary, return the child to bed. Keep the house tidy and safe
			REM parasomnias	Nightmare disorder	Reassuring measures; it may be useful to use a night light or a comfort object. Avoid exposure to anxiogenic stimuli, especially before bedtime. During the day, talk about the nightmare in order to “comfort” the child
			Other parasomnias	Sleep enuresis	Water restriction, try to empty the bladder before bed, child empowerment, involve the child in bed hygiene, don't judge. After the age of 7, investigation is warranted to exclude secondary cause enuresis
Sleep-related breathing disorders	Obstructive sleep apnea, pediatric, primary sleep apnea of infancy, apnea of prematurity	Treatment will depend on the cause. If the cause is overweight, the treatment will involve changes in diet and physical exercise. If the cause is a structural change or tonsil/adenoid hypertrophy, treatment may require surgical correction
Circadian rhythm sleep disorders	Delayed sleep phase type, irregular sleep-wake type	Intervention may include psychoeducation and cognitive-behavioral techniques. In some cases, the intervention of a psychiatrist may be necessary
Hypersomnias of central origin	Narcolepsy (type 1 and 2), idiopathic hypersomnia, secondary hypersomnia	Scheduled naps, pharmacological therapy, investigation to exclude an organic cause
Sleep movement disorders	Restless legs syndrome, bruxism, benign sleep myoclonus, rhythmic movements when falling asleep	Sleep hygiene, assessing iron deficiency, behavioral therapy, accident prevention, reassuring parents

Bed sharing and child sleep

Currently, the practice of bed-sharing, where the child and caregiver share the same sleeping surface, has been the subject of intense debate among parent groups and the medical community [25]. This is a topic often addressed in medical consultations, as many families eventually adopt the shared bedding model at some point in the child's life cycle.

Some studies have suggested that up to half of deaths from sudden infant death syndrome (SIDS) occur when babies sleep next to an adult [26,27]. This led some countries to advise against bed-sharing, including the American Academy of Pediatrics [26,27]. However, in these studies, the definition of “bed-sharing deaths” varied widely, encompassing sofa sharing, sharing surfaces with pets, and both situations in which bed-sharing was a family routine and situations in which it merely happened punctually [26,27]. Furthermore, not all studies took into account the circumstances in which bed-sharing occurred, namely babies who slept next to parents who smoked or drank alcohol [26,27].

It has been found that the majority of families that share beds are families in which the mother breastfeeds the baby [27]. Bed sharing facilitates nighttime breastfeeding and is associated with more frequent nighttime feedings (which promote milk production by taking advantage of the physiological peak of prolactin during the night), higher rates of exclusive breastfeeding, and continued breastfeeding for longer periods of time [27].

Bed sharing also has benefits in terms of preventing potentially fatal events through maternal and baby physiological mechanisms [25]. For example, compared to breastfed babies who sleep alone, breastfed babies who co-sleep spend less time in stages 3-4 (deep sleep) and more time in stages 1-2 (lighter sleep), making it easier for the rapid awakening of the baby and the cessation of apneas, preventing fatal events [25]. Polysomnographic studies comparing mothers who exclusively breastfeed and share a bed with mothers whose babies sleep on a separate surface show that, even in the deepest stages of sleep, mothers wake up 30% more frequently when sharing a bed [25]. Approximately half of maternal awakenings overlap with the baby's awakenings, and, in approximately two-thirds of these cases, the baby is awakened first, which suggests relatively high responsiveness on the part of the mother [25]. This sensitivity can increase the likelihood of mothers detecting and intervening quickly in the event of a life-threatening event for their baby [25].

Since breastfeeding has been associated with a reduction in the risk of SIDS, the prohibition of bed-sharing by health professionals could lead to weaning, with the consequent loss of a strong protective factor against SIDS [27]. Furthermore, such a ban could evoke fear of sleeping on the safe surface of the bed and put families at risk of getting out of bed to breastfeed their baby on a chair or sofa and potentially falling asleep there, a significantly more unsafe place than the bed [27].

When analyzing the number of child deaths in shared beds, it appears that factors such as maternal smoking, use of alcohol or drugs, sleeping on sofas as a common surface, and sleeping with pillows or under duvets substantially increase the risk of SIDS or fatal accidents in a shared bed [27-29].

There are several reasons that may lead to choosing a shared bed [26-29]. If this is the option desired by a given family, the family doctor must know how to advise and explain the care to be taken so that this is a safe practice [26-29]. Particular attention should be paid to the following indications [26-29]: the mattress must be firm and smooth; water or air mattresses are not suitable; the room should not be too hot (ideal temperature between 18 and 22 ºC); the baby should not be overdressed and should not have more clothes than they would have if they were in bed alone; other children or pets should not share the baby's bed; pay attention to the bed and the surrounding environment so that the baby cannot fall out of bed or become trapped between the mattress and the wall; blankets must not heat the baby too much or cover their head; and the baby cannot be left alone in bed.

Furthermore, the family doctor must be aware of the contraindications to bed-sharing, namely [26-29], sharing a bed with someone other than the baby's mother or father; a caregiver who uses medications with sedative potential (antidepressants, anxiolytics, hypnotics, antihistamines or muscle relaxants); caregiver who uses substances such as alcohol or drugs (even if they do not use them in bed); obese caregiver; caregiver with a high degree of exhaustion; caregiver who smokes (even if he does not smoke in bed) or has been an ex-smoker for less than one year; premature or low birth weight baby; child under four months (even without other risk factors); unsuitable mattress, for example, too soft or old, sofas or armchairs; and use of accessories in bed (pillows, diapers, toys, etc).

Family physician and proximity to families

One of the concerns of families in the early stages of a baby's life is sleep, which is why it is a frequently addressed topic in child health consultations [3,6]. Parameters such as the number of hours the baby sleeps; how he falls asleep; sleep cycles; characteristics of the crib, if the baby sleeps in their own crib; room temperature; and safety issues during sleep to reduce the risk of SIDS are discussed [3,6]. Parents often seek clarification from their family physician to ensure the best approach during this period of their lives and their children's lives [3,6].

Throughout life, we should be concerned with the number of hours of sleep as well as its quality and its impact on our daily lives [3,6,7]. It is important to understand how the family relates to sleep, what rituals are associated with bedtime, and to explain that the number of hours of sleep and sleep cycles vary with age, with the need for hours of sleep varying over the course of life for each person [3,6,7].

According to the Portuguese National Program for Child and Adolescent Health, issued by the Direção Geral da Saúde, the topic “sleep” should be addressed in all consultations [24]. Sleep disorders in pediatrics are common, and unlike in adulthood, they will affect not only the child's development but also the dynamics of the entire family [3,6,7]. Therefore, it is important for family physicians to have the appropriate tools to address this issue and, if necessary, refer to and/or treat it.

Family physicians can thus be a key factor in improving the health quality of families and caregivers, as they are generally the first medical contact within the healthcare system [3,6,7,24,30]. By developing a person-centered approach oriented toward the individual and their family, they can contribute to improving the quality of life for all family members [3,6,7,24,30]. It is important to note that by providing longitudinal continuous care, they promote the child's follow-up through various stages of life, always keeping abreast of changes or problems that may arise [3,6,7,24,30]. It is also important to emphasize that family physicians adopt a holistic approach, having the ability to use a biopsychosocial model and taking into account cultural and existential dimensions [3,6,7,24,30].

## Conclusions

Sleep plays a fundamental role in various areas: neurodevelopment, behavior, growth, and performance. Therefore, it is imperative that family physicians are sensitive to this issue, as they are in a privileged position to assist families given their proximity and knowledge of their context.

In general practice and family medicine consultations, it is crucial to investigate the sleep routines and rituals of children to ensure adequate rest. Therefore, it is essential to invest in health literacy, inform parents, and encourage children to adopt good health practices, thus making sleep one of the central topics in child health consultations.
